# Development of Novel Prenatal Prediction Models for Birth-Weight Estimation in Low-Risk Third-Trimester Pregnancies

**DOI:** 10.3390/biomedicines14092122

**Published:** 2026-09-19

**Authors:** Sevda Yelec, Rauf Melekoglu, Yusuf Kemal Arslan, Cihan Cetin, Selim Buyukkurt, Gulsah Seydaoglu

**Affiliations:** 1 Department of Obstetrics and Gynecology, Diyarbakır Gazi Yasargil Health Research Center, University of Health Sciences, Diyarbakır 21070, Türkiye; 2 Department of Obstetrics and Gynecology, Faculty of Medicine, Inonu University, Malatya 44280, Türkiye; 3 Department of Biostatistics, Faculty of Medicine, Cukurova University, Adana 01330, Türkiye; ykarslan@gmail.com (Y.K.A.); gseydaoglu@gmail.com (G.S.); 4 Department of Obstetrics and Gynecology, Faculty of Medicine, Bahcesehir University, Istanbul 34353, Türkiye; cihancetin00@yahoo.com; 5 Department of Obstetrics and Gynecology, Faculty of Medicine, Cukurova University, Adana 01330, Türkiye; selimbuyukkurt@gmail.com

**Keywords:** estimated fetal weight, regression analysis, soft-tissue indices, third trimester, ultrasound

## Abstract

**Objective:** Sonographic evaluation of the fetus and estimation of fetal weight are integral components of modern obstetric practice. Estimated fetal weight (EFW) plays a crucial role in predicting macrosomia, diagnosing intrauterine growth restriction, and monitoring fetal development. This study aimed to improve birth-weight prediction in low-risk term pregnancies by combining conventional fetal biometry with maternal anthropometry and selected fetal soft-tissue and cardiac measurements. **Materials and Methods:** In 94 low-risk, term singleton pregnancies scheduled for elective cesarean delivery, standardized prenatal ultrasound was performed within 24 h before birth by a single operator. Neonatal anthropometry was obtained within 6 h postnatally. Multiple linear regression was used to develop conventional and extended prenatal birth-weight prediction models with actual birth weight as the dependent variable, and their performance was compared with Hadlock EFW. **Results:** Mean actual birth weight was 3178.9 ± 359.5 g and mean Hadlock EFW was 3201.6 ± 399.7 g. Hadlock estimation correlated strongly with birth weight (r = 0.826) and had a mean absolute error (MAE) of 181.0 g. Abdominal circumference was the strongest conventional sonographic correlate of birth weight. The conventional prenatal Model 2 achieved R^2^ = 0.710 and MAE = 150.1 g. The extended prenatal Model 5, which combined conventional biometry with symphysis-fundal height, femoral soft-tissue thickness, right atrial area, maternal abdominal fat thickness, and aortic valve diameter, showed the best performance (R^2^ = 0.783; MAE = 133.2 g). Its optimism-corrected and repeated cross-validated R^2^ values were 0.739 and 0.725, respectively. **Conclusions:** The newly proposed formulas, particularly Model 5, demonstrated better internally validated predictive performance than the Hadlock baseline in this cohort, suggesting that the integration of selected maternal anthropometric and additional fetal ultrasonographic measurements with conventional biometry may improve birth-weight estimation. These findings warrant external validation in larger and more diverse populations prior to clinical implementation.

## 1. Introduction

Accurate estimation of fetal weight represents one of the cornerstones of modern obstetric practice, as it directly influences antenatal surveillance, the timing and mode of delivery, and perinatal outcomes [1]. Since the introduction of ultrasonography into obstetrics more than four decades ago, a multitude of biometric parameters and prediction models have been proposed to improve the precision of birth-weight estimation [2]. The clinical relevance of reliable estimated fetal weight (EFW) extends beyond academic interest; it plays a pivotal role in the antenatal diagnosis of intrauterine growth restriction (IUGR), the prediction of macrosomia, and the anticipation of delivery complications such as shoulder dystocia, birth trauma, or neonatal asphyxia [3,4]. Both underestimation and overestimation of fetal weight may result in adverse maternal and neonatal outcomes, including unnecessary operative interventions or failure to prevent high-risk deliveries.

The most widely accepted and applied formulas, such as those proposed by Hadlock and colleagues, combine conventional biometric parameters including biparietal diameter (BPD), head circumference (HC), abdominal circumference (AC), and femur length (FL). These formulas have achieved broad clinical applicability; however, their accuracy remains limited, with reported error rates ranging between 10 and 15% [5]. Importantly, this margin of error tends to increase in fetuses with extreme weights, either small for gestational age (SGA) or large for gestational age (LGA), which are precisely the groups in greatest need of accurate assessment [6]. Several studies have also highlighted that population differences, maternal characteristics, operator variability, and the time interval between ultrasound measurement and delivery can further compromise predictive accuracy [7,8].

In recent years, investigators have sought to refine EFW models by incorporating additional sonographic parameters, such as soft-tissue thickness, thigh or humeral skinfold, thoracic or abdominal fat measurements, and umbilical-cord cross-sectional area [9,10]. These innovative approaches reflect a shift from purely skeletal dimensions to combined assessments that include markers of fetal adiposity and body composition. Preliminary evidence suggests that such extended biometric profiles may better capture the heterogeneity of fetal growth patterns, thereby enhancing predictive performance over conventional formulas [11]. Nevertheless, despite the growing body of literature, no universally accepted model has yet achieved the desired level of accuracy in daily obstetric practice.

Given the clinical importance of precise fetal-weight estimation and the limitations of existing models, further research is warranted to develop and validate new formulas that integrate both conventional and novel biometric parameters. The present study was designed to address this gap by prospectively evaluating low-risk pregnancies in the third trimester and exploring the predictive value of multiple ultrasound-based measurements, including both traditional and less commonly utilized indices. Through regression modeling, we aimed to generate novel formulas that might surpass the predictive accuracy of widely used methods such as the Hadlock formula. Ultimately, the study seeks to contribute to improving perinatal management by providing more reliable tools for clinicians in the estimation of birth weight.

## 2. Materials and Methods

### 2.1. Study Design and Ethical Approval

This prospective observational study was conducted at the Department of Obstetrics and Gynecology, Cukurova University Faculty of Medicine, between 12 September 2013, and 2 May 2014. The study protocol was reviewed and approved by the Cukurova University Institutional Ethics Committee (5 July 2013, decision no. 21), and all procedures were performed in accordance with the Declaration of Helsinki and relevant national regulations. Each participant was informed verbally and in writing about the purpose and procedures of the study, and signed informed consent was obtained prior to enrollment. The study adhered to the Strengthening the Reporting of Observational Studies in Epidemiology (STROBE) guidelines.

### 2.2. Study Population

Pregnant women presenting for routine antenatal follow-up and scheduled for elective cesarean delivery at term were screened. Only low-risk, singleton pregnancies with a gestational age confirmed by a first-trimester crown-rump length measurement were considered for inclusion. Exclusion criteria were designed to eliminate conditions that could influence fetal growth or interfere with ultrasonographic measurements. Specifically, women with multiple pregnancies, intrauterine fetal demise, chromosomal or major structural anomalies, hydrops fetalis, placenta previa or accreta spectrum disorders, and oligohydramnios or polyhydramnios were excluded. In addition, maternal medical complications such as preeclampsia, gestational diabetes, chronic hypertension, renal disease, asthma, and autoimmune or thromboembolic disorders were considered exclusionary. After applying these criteria, 94 women were included in the final study cohort.

### 2.3. Prenatal Ultrasonographic Assessment

All participants underwent detailed ultrasonographic examination within 24 h before delivery. All examinations were performed by the same experienced obstetrician (S.B.) using a GE Voluson Pro ultrasound system (GE Medical Systems, Kretztechnik GmbH, Zipf, Austria) equipped with an RAB2-5L 2–5 MHz transabdominal transducer. To minimize interobserver variability, the same operator acquired all fetal measurements in accordance with the International Society of Ultrasound in Obstetrics and Gynecology (ISUOG) guidelines [6].

Conventional biometric parameters included BPD, HC, occipito-frontal diameter (OFD), AC, FL, and humerus length (HL). Abdominal transverse and anteroposterior diameters were obtained on the same transverse abdominal plane used for AC. Fetal abdominal subcutaneous tissue thickness was measured on this plane from the outer skin surface to the outer border of the anterior abdominal wall musculature. Femoral and humeral soft-tissue thicknesses were measured perpendicular to the long axis of the respective bone at the mid-diaphyseal level, from the outer skin surface to the bone cortex. Thoracic circumference was traced on a transverse four-chamber plane. Cardiac circumference, cardiac angle, chamber diameters and areas, valve diameters, and foramen ovale diameter were obtained from optimized four-chamber or outflow-tract views, as anatomically appropriate. The umbilical-cord diameter and total cross-sectional area were measured at a free loop using an outer-to-outer border trace; Wharton’s jelly area was calculated after subtracting the vascular areas. Placental thickness was measured perpendicular to the chorionic plate at the site of cord insertion. Standardized scanning planes and optimized gain settings were used for all measurements, with minimal abdominal pressure to avoid distortion. These exploratory measurements were included to represent complementary aspects of fetal size and body composition, including skeletal growth, truncal size, adipose deposition, cardiovascular dimensions, and fetoplacental tissue, based on previously reported associations of fetal soft tissue, thoracic dimensions, limb volume, and umbilical-cord biometry with fetal growth or birth weight.

Doppler velocimetry was also performed as part of the ultrasonographic assessment. The pulsatility indices (PI) of the middle cerebral artery (MCA), umbilical artery (UA), and bilateral uterine arteries (UtA) were recorded. Measurements were obtained with an insonation angle less than 30°, and a gate size adjusted to vessel lumen diameter. At least three consecutive uniform waveforms were recorded and averaged for each Doppler variable.

### 2.4. Maternal Measurements

Maternal anthropometry was conducted on the same day as ultrasonographic evaluation. Height and weight were recorded, and body mass index (BMI) was calculated as weight in kilograms divided by height in meters squared (kg/m^2^). Total gestational weight gain was determined by subtracting the first-trimester weight from the last recorded antenatal weight. Symphysis-fundal height was measured in the supine position using a non-stretchable measuring tape, from the superior border of the symphysis pubis to the highest palpable point of the uterine fundus. Maternal abdominal subcutaneous fat thickness was measured with a Harpenden-type skinfold caliper, placed 2 cm lateral to the umbilicus while the woman was standing in a relaxed posture. Each measurement was repeated three times, and the mean value was recorded to reduce intraobserver error.

### 2.5. Neonatal Measurements

Immediately after delivery, newborns were evaluated within 6 h by a single trained pediatric resident who was blinded to prenatal ultrasound findings. Birth weight was measured to the nearest 10 g using a calibrated electronic scale with the neonate unclothed. Crown-heel length was measured in the supine position with the head in the Frankfort plane and the lower limbs gently extended. Head circumference was measured over the occipital prominence and supraorbital ridges, thoracic circumference at the nipple line at the end of quiet expiration, and abdominal circumference at the umbilical level using a non-stretchable tape. Postnatal abdominal, femoral, and humeral skinfold thicknesses were measured with a Harpenden-type caliper at the same anatomical regions targeted prenatally: 2 cm lateral to the umbilicus and at the midpoints of the thigh and upper arm. Each anthropometric measurement was obtained twice and averaged. Actual birth weight was used as the reference outcome for regression modeling.

### 2.6. Statistical Analysis

All data were entered into a dedicated database and analyzed using IBM SPSS Statistics v20 (IBM Corp., Armonk, NY, USA). Continuous variables were tested for normality using the Shapiro–Wilk test. Normally distributed variables were expressed as mean ± standard deviation, and non-normally distributed variables were presented as median with interquartile range. Group comparisons were performed using independent-samples *t*-tests for normally distributed variables and Mann–Whitney U tests for non-parametric data. Categorical variables were summarized as counts and percentages and compared using chi-square or Fisher’s exact tests, as appropriate.

Pearson correlation analysis was used to examine associations between continuous prenatal measurements, Hadlock-estimated fetal weight, and actual birth weight. Multivariable linear regression was then performed with actual birth weight as the dependent variable. Candidate predictors comprised conventional biometric measurements, selected maternal anthropometric variables, and exploratory measurements representing fetal soft tissue, thoracic size, cardiac anatomy, umbilical cord, placenta, and Doppler velocimetry. Separate models were constructed to distinguish conventional prenatal prediction, extended prenatal prediction, reproduction of the Hadlock estimate, and postnatal explanatory benchmarks. Regression coefficients, standard errors, 95% confidence intervals, R^2^, and adjusted R^2^ were examined. Multicollinearity and residual assumptions were assessed using variance inflation factors and residual diagnostics.

Independent variables were originally selected using exploratory correlation screening followed by multivariable linear regression. The clinically relevant prenatal model specifications were fixed before validation: Model 2 included AC, BPD, and FL; Model 5 included symphysis-fundal height, BPD, AC, right atrial area, maternal abdominal skinfold thickness, FL, femoral skinfold thickness, and aortic valve diameter; and Model 6 included symphysis-fundal height, BPD, AC, right atrial area, FL, and femoral skinfold thickness. Each model was refitted in the *n* = 94 cohort. Model 1, which reproduced Hadlock-estimated weight from its component measurements, and Models 3 and 4, which used postnatal measurements, were retained only as methodological/explanatory analyses and were not treated as antenatal prediction models.

Model performance was assessed using R^2^, mean absolute error (MAE), root mean squared error (RMSE), mean signed percentage error, mean absolute percentage error (MAPE), and the proportion of predictions within ±10% of actual birth weight. Agreement was assessed using Bland–Altman mean bias and 95% limits of agreement. Percentile 95% confidence intervals were obtained from 5000 case-resampling bootstrap samples. Internal validation used 2000 bootstrap samples to estimate optimism-corrected R^2^, MAE, and RMSE. Stability was additionally examined using 100 repetitions of shuffled 10-fold cross-validation. Paired differences in absolute error between each prenatal model and Hadlock were evaluated using 5000 bootstrap resamples. All statistical tests were two-sided, and *p* < 0.05 was considered statistically significant.

### 2.7. Sample Size and Power Considerations

An a priori sample-size calculation was performed before recruitment for the planned multiple linear regression analysis. Assuming a medium overall effect size (Cohen’s f^2^ = 0.15), as defined by Cohen, a two-sided α level of 0.05, a statistical power of 80%, and five predictor variables, the minimum required sample size was calculated as approximately 92 participants [12]. Because the subsequent exploratory model-development analyses considered a broader set of candidate predictors than assumed in the original sample-size calculation, bootstrap optimism correction and repeated 10-fold cross-validation were additionally performed to assess model stability and potential overfitting.

## 3. Results

### 3.1. Baseline Characteristics

A total of 94 low-risk singleton pregnancies were included in the final analysis. Of the neonates, 50 (53.2%) were male and 44 (46.8%) were female. All included pregnancies had normal amniotic fluid indices. Fetal presentation was cephalic in 87 cases (92.6%) and non-cephalic in 7 (7.4%). Placental location was anterior in 34 pregnancies (36.2%) and posterior/fundal in 60 (63.8%). With respect to uterine scar history, 63 women (67.0%) had none or a single previous cesarean section, whereas 31 (33.0%) had two or more previous cesarean scars. The indication for cesarean delivery was previous cesarean delivery in 91 cases (96.8%), cephalopelvic disproportion in 2 (2.1%), and malpresentation without a previous cesarean delivery in 1 (1.1%).

Maternal anthropometric assessment showed a mean symphysis-fundal height of 37.4 ± 2.6 cm, mean abdominal subcutaneous fat thickness of 3.7 ± 1.0 cm, mean body mass index of 31.8 ± 5.0 kg/m^2^, and mean gestational weight gain of 11.3 ± 5.2 kg. Maternal baseline characteristics are summarized in Table 1.

### 3.2. Neonatal and Ultrasonographic Measurements

Mean actual birth weight was 3178.9 ± 359.5 g (median 3165 g; range 2230–4110 g). In comparison, the Hadlock formula estimated a mean fetal weight of 3201.6 ± 399.7 g (median 3170.9 g; range 2187.6–4257.6 g). The mean Hadlock prediction error, defined as estimated minus actual weight, was 22.7 ± 227.0 g.

Postnatal neonatal anthropometry showed mean head, abdominal, and thoracic circumferences of 348.24 ± 13.96 mm, 316.44 ± 19.61 mm, and 330.67 ± 15.01 mm, respectively. Mean neonatal abdominal, femoral, and humeral subcutaneous soft-tissue thicknesses were 4.32 ± 1.13 mm, 8.07 ± 2.40 mm, and 4.86 ± 1.14 mm, respectively. The mean postnatal umbilical-cord entry diameter was 3.54 ± 0.68 mm.

Prenatal ultrasonographic biometry yielded mean values of 90.70 ± 4.25 mm for BPD, 331.52 ± 16.09 mm for HC, 118.71 ± 6.17 mm for occipito-frontal diameter, 334.57 ± 19.20 mm for AC, 72.90 ± 3.71 mm for FL, and 62.69 ± 4.41 mm for HL. Mean prenatal abdominal, humeral, and femoral soft-tissue thicknesses were 3.88 ± 0.85 mm, 3.85 ± 1.18 mm, and 3.39 ± 0.94 mm, respectively. Umbilical-cord cross-sectional area was 168.71 ± 37.71 mm^2^, of which Wharton’s jelly accounted for 90.44 ± 24.23 mm^2^. Mean placental thickness was 33.76 ± 8.71 mm. Thoracic circumference was 272.46 ± 27.45 mm, with anteroposterior and transverse diameters of 91.47 ± 7.13 mm and 82.33 ± 7.87 mm. Mean heart angle, cardiac circumference, and aortic valve diameter were 44.19 ± 10.79°, 147.89 ± 14.29 mm, and 6.96 ± 0.92 mm. Right and left atrial areas were 286.74 ± 70.80 mm^2^ and 239.67 ± 44.95 mm^2^. Table 2 summarizes the distribution of prenatal ultrasonographic and postnatal anthropometric measurements; Table 3 presents their paired comparisons.

### 3.3. Correlation Between Ultrasound and Actual Neonatal Measures

**Figure 1:**
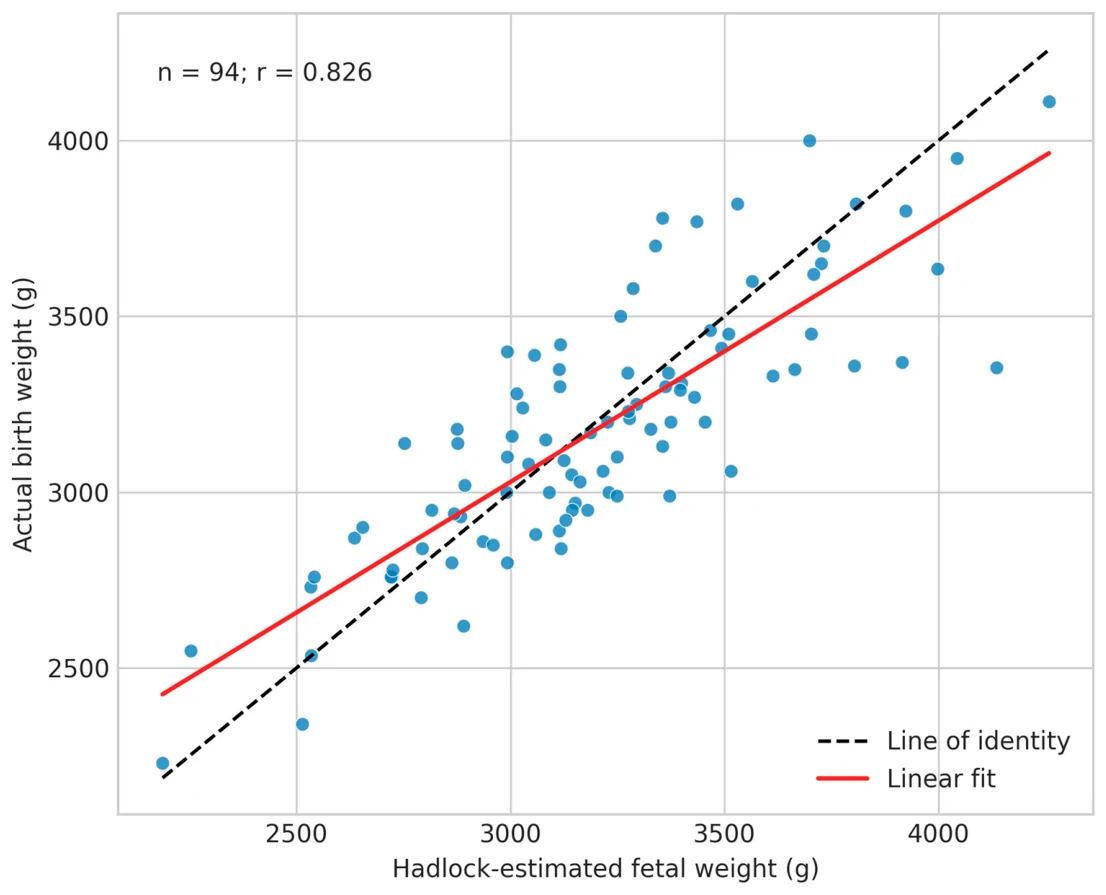
Linear regression of actual birth weight versus Hadlock-estimated fetal weight. Each blue dot represents an individual participant.

**Figure 2:**
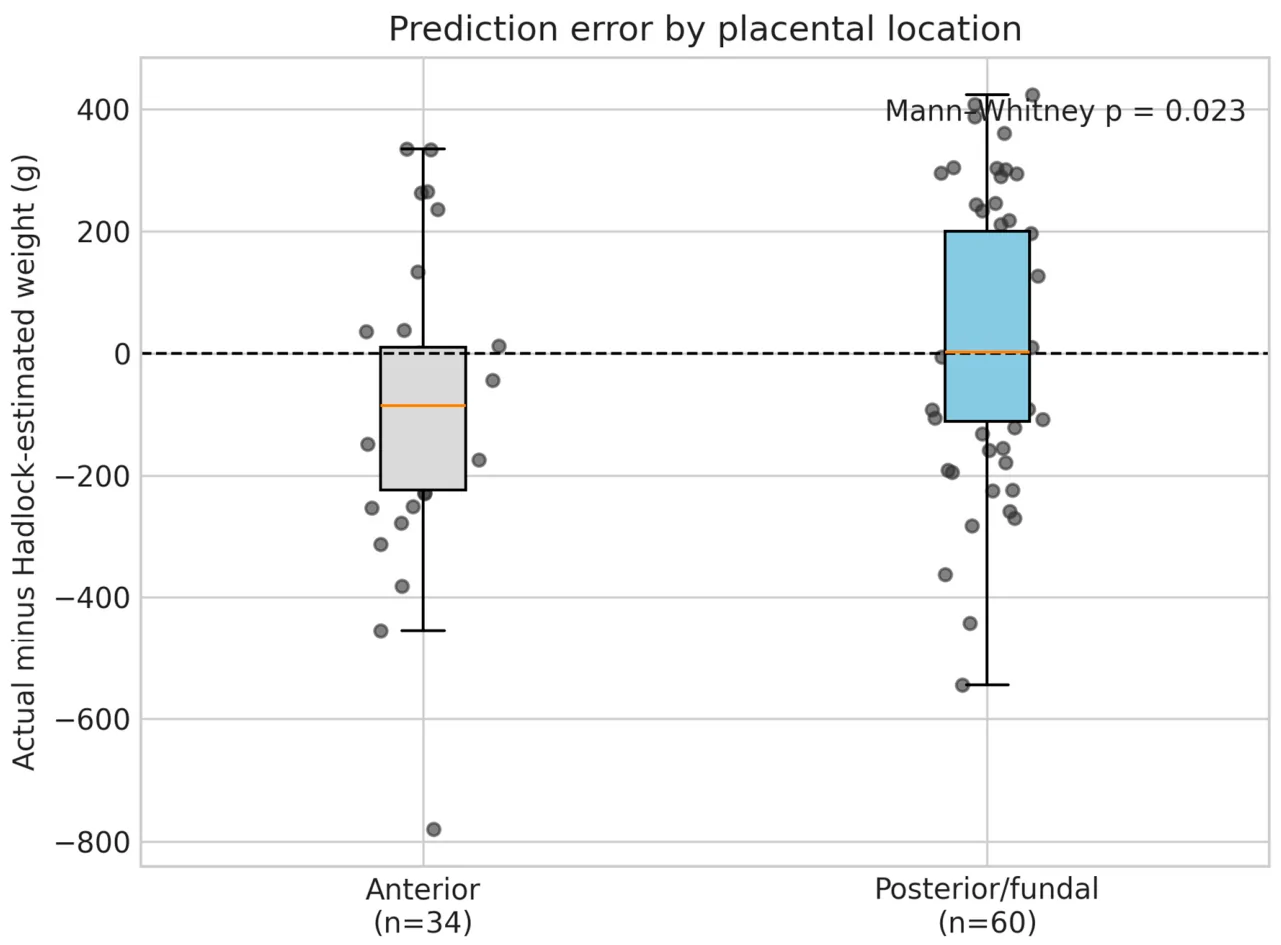
Actual-minus-Hadlock prediction difference by placental location. The orange horizontal line within each box represents the median, and individual data points represent individual participants. The dashed horizontal line indicates zero prediction error.

Hadlock-estimated fetal weight was strongly correlated with actual birth weight (r = 0.826, *p* < 0.001; Figure 1). The mean prediction error (estimated minus actual weight) was 22.7 g, with 95% limits of agreement from −422.3 to 467.7 g. The MAE was 181.0 g, RMSE was 227.0 g, mean absolute percentage error was 5.67%, and 89.4% of estimates were within ±10% of actual birth weight. Subgroup analysis revealed no significant difference in Hadlock prediction error according to cesarean scar number (*p* = 0.717) or fetal presentation (*p* = 0.621), whereas an unadjusted difference was observed according to placental location (*p* = 0.023). (Table 4 and Figure 2).

Comparisons between corresponding prenatal ultrasonographic and postnatal anthropometric measurements demonstrated moderate positive correlations for abdominal (r = 0.458) and femoral (r = 0.453) soft-tissue thicknesses, whereas the correlation for humeral soft-tissue thickness was weaker (r = 0.324). Among the conventional anthropometric measurements, HC showed the strongest prenatal–postnatal correlation (r = 0.646), followed by AC (r = 0.511), while substantially weaker correlations were observed for thoracic circumference (r = 0.160) and umbilical-cord entry diameter (r = 0.224). In addition, ultrasound measurements of the umbilical-cord entry diameter were significantly larger than the corresponding postnatal measurements (7.06 ± 1.52 vs. 3.54 ± 0.68 mm; *p* < 0.001).

When the associations with actual birth weight were examined, postnatal thoracic circumference showed the strongest correlation (r = 0.760), followed by postnatal AC (r = 0.656). Among prenatal measurements, ultrasound-derived abdominal circumference was the strongest correlate of actual birth weight (r = 0.747) and also showed a particularly strong correlation with Hadlock-estimated fetal weight (r = 0.883). Other prenatal measurements showing notable correlations with actual birth weight included BPD (r = 0.605), symphysis-fundal height (r = 0.488), HC (r = 0.442), and FL (r = 0.409). Complete univariable correlations of actual birth weight and Hadlock-estimated fetal weight with the candidate predictors, together with the correlations between prenatal and corresponding postnatal measurements, are provided in Supplementary Tables S1 and S2.

### 3.4. Regression Modeling

Multiple regression analyses were performed to develop and compare models for birth-weight estimation:

**Model 1 (Hadlock reconstruction model):** Using AC, FL, HC, and BPD, the model reconstructed Hadlock-estimated fetal weight with an R^2^ of 0.994. This model served as a reconstruction check rather than as a new model for predicting actual birth weight.

**Model 2 (conventional prenatal model):** The combination of AC, BPD, and FL explained 71.0% of the variance in actual birth weight (R^2^ = 0.710), with an MAE of 150.1 g and an RMSE of 192.7 g.

**Model 3 (postnatal neonatal model):** The model incorporating neonatal thoracic circumference, neonatal length, humeral and femoral soft-tissue thicknesses, and AC explained 82.2% of the variance in actual birth weight (R^2^ = 0.822). Because all predictors were obtained after delivery, this model was retained only as an explanatory benchmark and was not considered applicable to antenatal prediction.

**Model 4 (postnatal neonatal model excluding length):** Thoracic circumference, femoral and humeral soft-tissue thicknesses, HC, and AC explained 73.2% of the variance in actual birth weight (R^2^ = 0.732). Similar to Model 3, this model was retained only as an explanatory benchmark.

**Model 5 (extended prenatal model):** The combination of symphysis-fundal height, BPD, AC, right atrial area, maternal abdominal fat thickness, FL, femoral soft-tissue thickness, and aortic valve diameter provided the best performance among the prenatal models (R^2^ = 0.783; MAE = 133.2 g; RMSE = 166.6 g).

**Model 6 (reduced extended prenatal model):** A more compact combination of symphysis-fundal height, BPD, AC, right atrial area, FL, and femoral soft-tissue thickness explained 74.6% of the variance in actual birth weight (R^2^ = 0.746), with an MAE of 146.1 g and an RMSE of 180.3 g.

Among the models predicting actual birth weight, Model 3 showed the highest apparent explanatory performance; however, because it relied exclusively on postnatal measurements, it has no antenatal predictive application. Among the clinically relevant prenatal models, Model 5 showed the best apparent performance. Internal validation resulted in optimism-corrected/repeated cross-validated R^2^ values of 0.693/0.683 for Model 2, 0.739/0.725 for Model 5, and 0.713/0.699 for Model 6, indicating that Model 5 retained the highest predictive performance after correction for optimism and repeated cross-validation.

**Figure 3:**
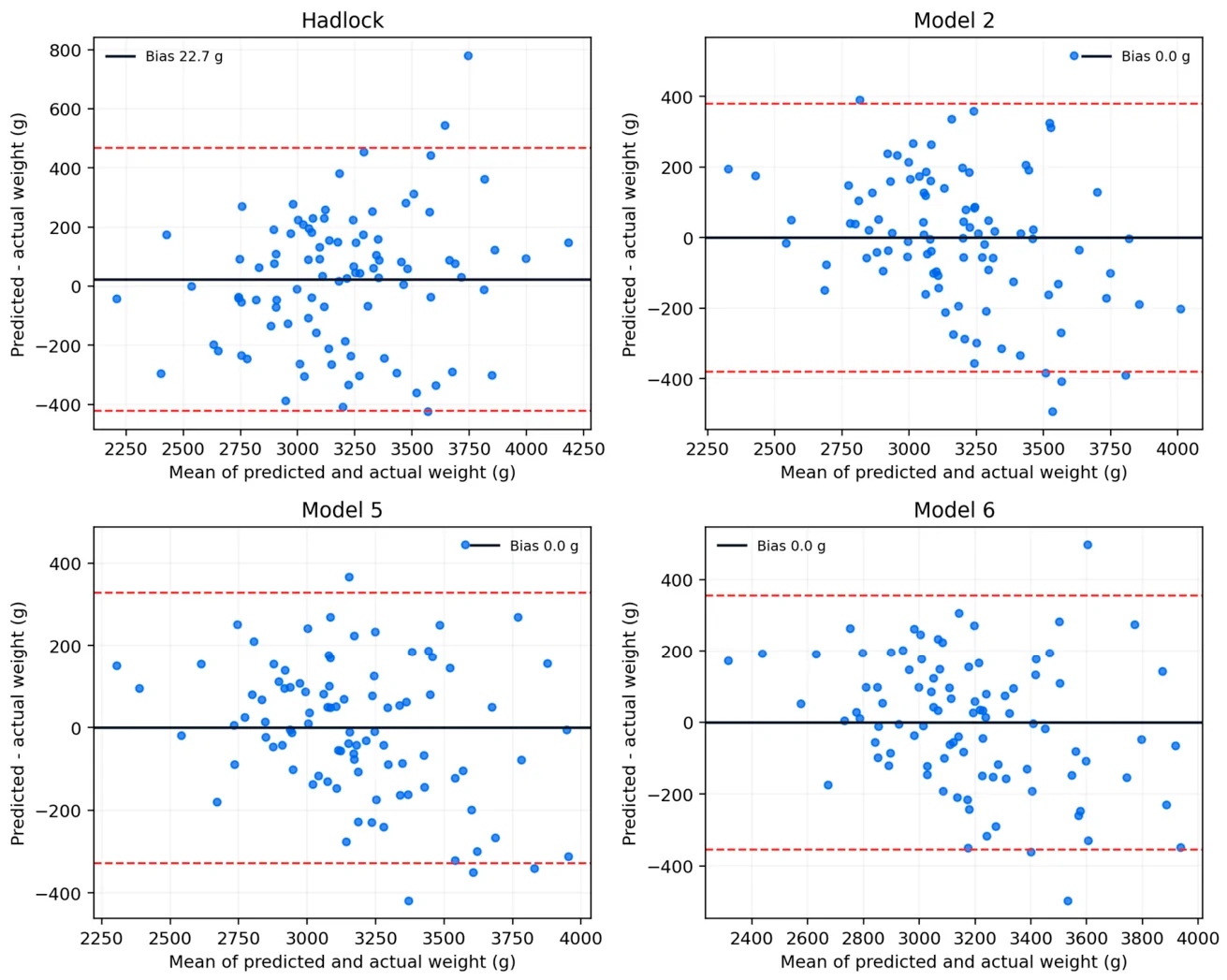
Bland–Altman agreement plots for Hadlock and the three prenatal prediction models. Solid lines indicate mean bias and red dashed lines indicate 95% limits of agreement.

**Figure 4:**
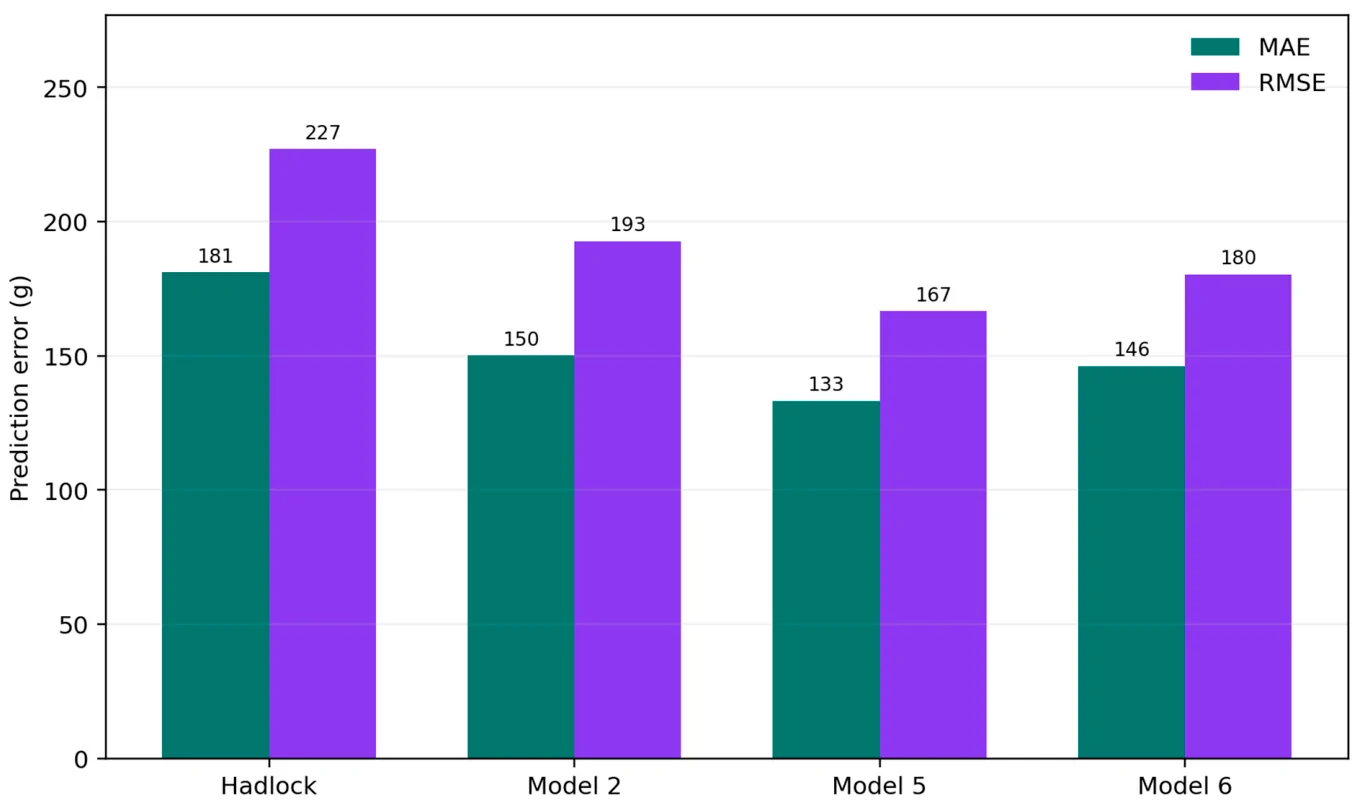
Comparison of apparent mean absolute error (MAE) and root mean squared error (RMSE) for Hadlock estimation and the three prenatal prediction models (Models 2, 5, and 6).

Compared with Hadlock estimation, the MAE was reduced by 30.9 g for Model 2 (bootstrap 95% CI, 11.1–50.6 g), 47.8 g for Model 5 (95% CI, 22.8–72.9 g), and 34.9 g for Model 6 (95% CI, 13.0–57.4 g). For Model 5, 91 of 94 predictions (96.8%) were within ±10% of actual birth weight. Regression equations and model performance are summarized in Table 5 and Table 6, while agreement and error comparisons are presented in Figure 3 and Figure 4.

## 4. Discussion

Accurate estimation of fetal weight remains one of the most important challenges in obstetric practice, as it directly influences antenatal surveillance, decisions regarding the timing and mode of delivery, and strategies to reduce maternal and neonatal morbidity. Although numerous formulas based on conventional biometric parameters such as BPD, HC, AC, and FL have been developed, no single approach provides consistently optimal accuracy across different populations and fetal-weight categories [13,14]. The performance of these equations may be influenced by biological variation in fetal growth and body composition, maternal characteristics, pregnancy-related conditions, population differences, and technical factors related to image acquisition and measurement. Against this background, the present study investigated whether maternal anthropometric characteristics and additional fetal soft-tissue and cardiac measurements could provide complementary information beyond conventional fetal biometry and improve the estimation of actual birth weight.

In the present study, we deliberately restricted the cohort to low-risk, term singleton pregnancies delivered by elective cesarean section and standardized the timing and acquisition of ultrasonographic measurements. This design reduced heterogeneity related to pathological fetal growth and minimized the interval between prenatal ultrasound and actual birth-weight measurement. Within this closely standardized cohort, abdominal circumference emerged as the strongest conventional sonographic correlate of actual birth weight (r = 0.747), consistent with its established importance as a marker of fetal size and soft-tissue growth. Hadlock-estimated fetal weight also showed a strong association with actual birth weight; however, correlation alone did not fully reflect individual prediction error. The Hadlock approach had an MAE of 181.0 g and an RMSE of 227.0 g, whereas the extended prenatal Model 5 achieved lower corresponding errors of 133.2 g and 166.6 g. Model 5 incorporated conventional fetal biometry together with symphysis-fundal height, femoral soft-tissue thickness, maternal abdominal fat thickness, right atrial area, and aortic valve diameter, and remained the best-performing prenatal model after internal validation, with an optimism-corrected R^2^ of 0.739 and a repeated cross-validated R^2^ of 0.725. These findings suggest that measurements reflecting maternal characteristics and fetal soft-tissue accretion may provide complementary information to conventional skeletal and abdominal biometric dimensions. 

The limitations of conventional fetal-weight equations become particularly important at the extremes of fetal size, where clinically relevant underestimation or overestimation may influence decisions regarding surveillance and delivery [15]. Previous studies have therefore explored population-specific and condition-specific approaches to improve fetal-weight estimation. Chen et al. reported improved performance of population-specific models for low-birth-weight and macrosomic infants [16], whereas Faschingbauer et al. developed an equation specifically designed for extreme macrosomia [17]. In contrast, the present study was intentionally confined to low-risk term pregnancies rather than fetuses with growth restriction or macrosomia. This restriction provided a relatively homogeneous setting in which to examine the contribution of additional anthropometric and ultrasonographic measurements, but it also limits extrapolation of the resulting models to pregnancies at the extremes of fetal growth. Importantly, even within this selected population, the Hadlock MAE of 181.0 g and the observed limits of agreement demonstrate that a strong overall association between estimated and actual birth weight does not necessarily translate into equally accurate prediction at the individual level.

An important finding of our study was the contribution of maternal symphysis-fundal height and abdominal adiposity, parameters that are often omitted from conventional ultrasonographic fetal-weight equations. In the present analysis, symphysis-fundal height was retained in both extended prenatal models, while maternal abdominal fat thickness contributed to the more comprehensive Model 5. These findings suggest that maternal anthropometric characteristics may provide complementary information on the intrauterine growth environment that is not fully captured by fetal biometry alone. Indeed, Model 6, which integrated symphysis-fundal height with conventional fetal biometric parameters (BPD, AC, and FL), femoral soft-tissue thickness, and right atrial area, achieved an apparent R^2^ of 0.746, with an MAE of 146.1 g and an RMSE of 180.3 g. The more comprehensive Model 5, which additionally incorporated maternal abdominal fat thickness and aortic valve diameter, showed the best performance among the prenatal models, with an apparent R^2^ of 0.783, an MAE of 133.2 g, and an RMSE of 166.6 g. Importantly, the relative performance of these extended models was largely maintained after internal validation, with optimism-corrected/repeated cross-validated R^2^ values of 0.713/0.699 for Model 6 and 0.739/0.725 for Model 5. These observations are consistent with the hybrid modeling approach reported by Nahum and colleagues, who incorporated maternal characteristics such as height and weight alongside fetal measurements to improve birth-weight estimation [18]. Taken together, our findings support further investigation of models combining maternal anthropometry with conventional fetal biometry and selected additional ultrasonographic measurements as a potentially more comprehensive approach to prenatal birth-weight estimation.

The role of fetal soft-tissue parameters in refining birth-weight estimation is increasingly recognized, as conventional skeletal biometry alone may not fully capture the variability in fetal body composition that ultimately contributes to birth weight [19]. In our analysis, femoral soft-tissue thickness provided complementary predictive information beyond conventional biometry and was retained in both extended prenatal models (Models 5 and 6). This finding is consistent with previous studies by Catalano et al. and Lee et al., which highlighted fetal adiposity as an important determinant of neonatal body mass and growth and demonstrated its relationship with maternal metabolic characteristics and maternal-placental nutrient availability [20,21]. Taken together, these observations support the concept that variation in soft-tissue accretion may account for part of the birth-weight variability not fully represented by conventional skeletal and abdominal biometric measurements. Our findings further suggest that information beyond fetal adiposity may contribute to extended prediction models. Right atrial area was retained in both Models 5 and 6, while aortic valve diameter was additionally incorporated into Model 5. These cardiac dimensions may partly reflect overall fetal size and hemodynamic adaptation and may therefore provide information complementary to conventional fetal biometry. However, their specific incremental contribution was not evaluated independently in the present study, and their potential predictive value should be balanced against the additional acquisition time, technical expertise, and reproducibility requirements associated with these measurements.

While prenatal thoracic circumference showed only a weak correlation with actual birth weight in our cohort (r = 0.160), neonatal thoracic circumference demonstrated a substantially stronger association (r = 0.760). This marked discrepancy suggests that thoracic dimensions may contain relevant information on neonatal body size that is not captured with comparable precision by conventional two-dimensional prenatal ultrasonography. One possible explanation is the technical difficulty of consistently defining and measuring the fetal thoracic outline antenatally, rather than an absence of biological relevance of thoracic size itself. Similar technical considerations have been discussed in previous studies examining thoracic and chest circumference as adjunct measures of neonatal size and low birth weight [22,23,24]. The discrepancy observed in our study also highlights a broader challenge in translating fetal soft-tissue and body-composition measurements into reproducible prenatal predictors. Advances in three-dimensional ultrasonography and volumetric assessment, as reported by Wu, Lee, and Grantz, may help address this limitation by allowing more standardized and potentially more reproducible quantification of fetal limb and truncal dimensions [25,26,27]. Such approaches may ultimately improve the antenatal characterization of fetal body composition and help narrow the discrepancy between prenatal measurements and their corresponding postnatal anthropometric measures observed in the present study.

The subgroup findings were secondary to the main prediction analysis. Hadlock prediction error did not differ according to fetal presentation or cesarean-scar category. In contrast, an unadjusted difference in prediction error was observed according to placental location. Placental position may potentially influence the technical acquisition of fetal biometric measurements by altering fetal accessibility, insonation angle, or image optimization, although the present study was not designed to investigate these mechanisms. Importantly, placental location was not included as a predictor in the developed equations, and the observed association arose from a secondary subgroup analysis with relatively small group sizes and multiple comparisons. Therefore, this finding should be considered exploratory rather than evidence of an independent effect of placental location on fetal-weight estimation and warrants confirmation in larger cohorts.

Internal validation was performed to assess the extent to which the apparent performance of the prenatal models might reflect optimism related to model development in a relatively small cohort. Although the predictive performance of all models decreased after bootstrap correction and repeated cross-validation, Model 5 remained the best-performing prenatal model, with an apparent R^2^ of 0.783, an optimism-corrected R^2^ of 0.739, and a repeated cross-validated R^2^ of 0.725. The relatively modest reduction in performance suggests that the observed advantage of Model 5 was not solely attributable to in-sample optimism. Nevertheless, given the modest sample size and the number of candidate predictors considered during model development, some degree of residual overfitting and coefficient instability cannot be excluded. Accordingly, these internally validated results should be regarded as evidence supporting further evaluation of the model rather than confirmation of its generalizability, which requires external validation in an independent cohort.

Fetal growth is not a uniform biological process but is influenced by a complex interplay of genetic, ethnic, nutritional, maternal, and socioeconomic factors that may vary substantially between populations. Gardosi et al. demonstrated that customized growth standards incorporating maternal characteristics can improve differentiation between constitutionally small or large fetuses and those with pathological deviations in growth, while Buck Louis et al. showed, in a large multiethnic cohort, that fetal growth trajectories differ across racial and ethnic groups even after accounting for maternal characteristics [28,29]. These observations are particularly relevant to prediction models incorporating maternal anthropometric and fetal soft-tissue measurements, because both the regression coefficients and the relative contribution of individual predictors may vary across populations. The comparatively high mean maternal body mass index in our cohort may also be relevant, as maternal adiposity can affect the technical acquisition and reproducibility of some ultrasonographic measurements. Because the present study included only Turkish women recruited from a single tertiary center, the numerical coefficients and predictive performance of the proposed models should not be assumed to be directly transportable to other populations. External validation and, if necessary, recalibration in cohorts with different ethnic, anthropometric, nutritional, and obstetric characteristics will therefore be essential before broader clinical application.

## 5. Strengths and Limitations

The strengths of our study include its prospective design, strict inclusion criteria restricted to low-risk term pregnancies, the short interval between ultrasonographic assessment and delivery, standardized sonographic protocols, and the comprehensive evaluation of both conventional and additional maternal and fetal parameters. All prenatal ultrasonographic measurements were performed by a single experienced operator (S.B.), thereby minimizing interobserver variation during data acquisition. Moreover, neonatal anthropometric assessments were performed shortly after birth by a single examiner who was blinded to the prenatal measurements, providing standardized postnatal outcome assessment. Restriction of the cohort to pregnancies undergoing elective cesarean delivery further reduced potential variability related to labor and allowed prenatal measurements obtained within 24 h before delivery to be compared with actual birth weight over a closely standardized time interval. Another strength is the broad phenotypic assessment, which enabled conventional fetal biometry to be evaluated alongside maternal anthropometry, fetal soft-tissue thickness, thoracic and umbilical-cord measurements, and selected cardiac dimensions. Finally, the performance of the clinically relevant prenatal models was assessed using multiple complementary measures, including R^2^, MAE, RMSE, percentage-based error metrics, and agreement analysis, and model stability was further examined using bootstrap optimism correction and repeated 10-fold cross-validation.

Several limitations should nevertheless be acknowledged. First, the study was conducted at a single tertiary center in a relatively modest and highly selected cohort, which limits the external generalizability of the findings. Although restriction to low-risk term pregnancies increased cohort homogeneity and minimized several potential sources of measurement variability, the resulting models cannot be assumed to perform similarly in preterm pregnancies, pregnancies complicated by diabetes or fetal growth restriction, or fetuses at the extremes of the weight distribution, including SGA, LGA, and macrosomic fetuses. These populations are particularly important because accurate fetal-weight estimation may have its greatest clinical consequences at the extremes of fetal growth. Therefore, evaluation of the proposed models in these clinically relevant populations will be necessary before broader application can be considered. Second, the number of candidate predictors examined during model development was relatively large in relation to the final sample size (*n* = 94), particularly for the more complex Model 5, which included eight predictors. This predictor-to-sample-size relationship increases the risk of model overfitting and instability of individual regression coefficients. We attempted to address this limitation by applying bootstrap optimism correction and repeated 10-fold cross-validation to the clinically relevant prenatal models. Model 5 retained the highest performance after these procedures, with an optimism-corrected R^2^ of 0.739 and a repeated cross-validated R^2^ of 0.725; nevertheless, internal validation cannot substitute for evaluation in an independent population, and some optimism or coefficient instability may remain. Accordingly, the present models should be regarded as developmental prediction models requiring external validation rather than as equations ready for clinical implementation. Third, all ultrasonographic measurements were obtained by a single experienced operator. Although this approach reduced measurement variability within the present study, it precluded assessment of interobserver reproducibility and may have resulted in better measurement consistency than would be expected in routine clinical practice. This consideration is particularly relevant to the additional soft-tissue and cardiac measurements, which are less standardized than conventional fetal biometry and may require additional acquisition time, operator training, and technical expertise. The feasibility and reproducibility of these measurements should therefore be specifically evaluated across different operators and clinical settings. In addition, the present study relied predominantly on two-dimensional ultrasonography; three-dimensional and volumetric techniques may provide more reproducible characterization of fetal soft-tissue and body composition and warrant evaluation in future studies. Finally, although the present revision substantially expands the assessment of predictive performance beyond R^2^ by incorporating absolute and percentage-based prediction errors, Bland–Altman agreement analysis, bootstrap confidence intervals, optimism correction, and repeated cross-validation, external validation was not available. The apparent performance estimates should therefore continue to be interpreted cautiously, particularly for the more complex models. Larger, multicenter studies with independent validation cohorts are required to evaluate model transportability, interobserver reproducibility, calibration, and performance across different fetal-weight categories and maternal risk profiles before these models can be considered for clinical implementation.

### Clinical Implications and Future Directions

Our findings suggest that, while the Hadlock formula remains a valuable and practical tool for routine fetal-weight estimation, predictive performance may be further improved by integrating selected maternal anthropometric and additional ultrasonographic parameters, particularly fetal soft-tissue measurements, with conventional biometry. The proposed extended models require additional measurements and were not intended to replace established fetal-weight formulas in routine obstetric practice. Rather, if externally validated, they may serve as complementary approaches in selected clinical settings, particularly in tertiary referral centers where comprehensive fetal ultrasonography is routinely performed and more precise estimation of fetal weight may contribute to clinical decision-making.

The potential clinical relevance of improved fetal-weight estimation is particularly evident when abnormal fetal growth is suspected, because estimated fetal size contributes to surveillance and delivery planning. Farina and colleagues recently demonstrated that estimated fetal-weight percentile and gestational age at the routine 36-week assessment both contribute to labor-related risk stratification in pregnancies with suspected LGA fetuses [30]. Although our study was not designed to evaluate LGA, SGA, or macrosomic fetuses specifically, these findings provide a rationale for evaluating the proposed models in pregnancies at the extremes of fetal growth. Future studies should therefore focus on external validation in larger, multicenter, and more diverse populations, together with assessment of interobserver reproducibility and the clinical feasibility of the additional measurements. Three-dimensional and volumetric ultrasonography may further improve soft-tissue assessment and should also be explored in future model development.

## 6. Conclusions

In this prospective study of low-risk, term singleton pregnancies, conventional fetal biometry remained strongly associated with actual birth weight, with AC emerging as the strongest conventional sonographic correlate. However, the incorporation of selected maternal anthropometric, fetal soft-tissue, and additional ultrasonographic measurements provided complementary predictive information beyond conventional biometry. Among the prenatal models, the extended Model 5 demonstrated the best overall performance, with lower MAE and RMSE than Hadlock estimation, and its relative performance was maintained after bootstrap optimism correction and repeated cross-validation. Model 6, which incorporated a more limited set of additional measurements, also showed improved prediction error compared with Hadlock while requiring fewer extended parameters.

These findings support the concept that birth-weight estimation may benefit from extending conventional fetal biometry to incorporate selected measures reflecting maternal characteristics and fetal soft-tissue accretion. Nevertheless, the proposed models were developed in a relatively small, highly selected cohort of low-risk term pregnancies and should therefore be regarded as developmental rather than clinically established prediction tools. Their performance cannot be extrapolated to preterm pregnancies, pregnancies complicated by diabetes or fetal growth restriction, or fetuses at the extremes of the weight distribution, including SGA, LGA, and macrosomic fetuses. In addition, the reproducibility and practical feasibility of the extended measurements across different operators and clinical settings remain to be established.

Further research should therefore focus on external validation in larger, multicenter, and more diverse populations, including pregnancies at increased risk of abnormal fetal growth. Assessment of interobserver reproducibility and the incremental contribution of individual extended measurements will also be important in determining whether the additional complexity of these models translates into clinically meaningful improvement. Future studies incorporating three-dimensional or volumetric ultrasonography may further refine the assessment of fetal soft-tissue and body composition. If externally validated and shown to be reproducible, such integrative approaches may ultimately contribute to more accurate prenatal birth-weight estimation and more informed obstetric decision-making.

## Figures and Tables

**Table 1:** Maternal and pregnancy baseline characteristics.

Variable	Category	*n*	%
Fetal sex	Male	50	53.2
	Female	44	46.8
Amniotic fluid index	Normal	94	100.0
Fetal presentation	Cephalic	87	92.6
	Non-cephalic	7	7.4
Placental location	Anterior	34	36.2
	Posterior/fundal	60	63.8
Cesarean scar count	0–1	63	67.0
	≥2	31	33.0
Cesarean-delivery indication	Previous cesarean delivery	91	96.8
	Malpresentation without previous cesarean	1	1.1
	Cephalopelvic disproportion	2	2.1
Continuous variable		Mean ± SD	Median (min–max)
Symphysis-fundal height (cm)		37.4 ± 2.6	37.0 (31–45)
Maternal abdominal subcutaneous fat thickness (cm)		3.7 ± 1.0	3.8 (1.2–5.8)
Body mass index (kg/m^2^)		31.8 ± 5.0	32.0 (21.3–47.9)
Gestational weight gain (kg)		11.3 ± 5.2	11.5 (0–26)

SD, standard deviation.

**Table 2:** Prenatal sonographic and neonatal anthropometric measurements.

Category	Variable	Mean ± SD	Median (Min–Max)
Neonatal measurements	Crown-heel length (cm)	48.83 ± 1.76	49.00 (42.00–54.00)
	Abdominal skinfold thickness (mm)	4.32 ± 1.13	4.00 (2.30–9.50)
	Femoral skinfold thickness (mm)	8.07 ± 2.40	8.00 (4.30–14.00)
	Humeral skinfold thickness (mm)	4.86 ± 1.14	5.00 (3.00–9.00)
	Head circumference (mm)	348.24 ± 13.96	350.00 (310.00–395.00)
	Abdominal circumference (mm)	316.44 ± 19.61	320.00 (260.00–360.00)
	Thoracic circumference (mm)	330.67 ± 15.01	330.00 (300.00–380.00)
	Umbilical-cord diameter at entry (mm)	3.54 ± 0.68	3.50 (2.00–5.00)
Head	BPD (mm)	90.70 ± 4.25	90.15 (81.50–104.50)
	HC (mm)	331.52 ± 16.09	333.00 (246.90–375.10)
	OFD (mm)	118.71 ± 6.17	118.70 (94.90–133.50)
Abdomen	AC (mm)	334.57 ± 19.20	333.85 (288.30–378.20)
	Abdominal skinfold thickness (mm)	3.88 ± 0.85	3.70 (2.10–6.40)
	Abdominal anteroposterior diameter (mm)	112.06 ± 10.17	112.60 (83.60–158.00)
	Abdominal transverse diameter (mm)	101.21 ± 8.43	99.90 (81.30–123.40)
Humerus	HL (mm)	62.69 ± 4.41	63.00 (35.90–74.50)
	Humeral skinfold thickness (mm)	3.85 ± 1.18	3.60 (2.30–8.30)
Femur	FL (mm)	72.90 ± 3.71	73.15 (62.90–80.90)
	Femoral skinfold thickness (mm)	3.39 ± 0.94	3.30 (2.00–6.30)
Umbilical cord	Cross-sectional area (mm^2^)	168.71 ± 37.71	165.00 (100.00–296.00)
	Wharton’s jelly area (mm^2^)	90.44 ± 24.23	88.00 (49.00–158.00)
	Diameter at entry (mm)	7.06 ± 1.52	6.90 (3.80–11.50)
Placenta	Thickness at cord insertion (mm)	33.76 ± 8.71	32.95 (2.75–57.50)
Thorax	Circumference (mm)	272.46 ± 27.45	274.90 (110.50–346.90)
	Anteroposterior diameter (mm)	91.47 ± 7.13	92.10 (76.40–107.50)
	Transverse diameter (mm)	82.33 ± 7.87	82.15 (65.90–103.60)
Heart	Heart angle (°)	44.19 ± 10.79	41.90 (20.80–75.80)
	Cardiac circumference (mm)	147.89 ± 14.29	144.65 (120.60–200.60)
	Tricuspid valve diameter (mm)	13.11 ± 1.62	13.10 (9.50–18.00)
	Mitral valve diameter (mm)	11.96 ± 2.01	11.90 (3.40–17.40)
	Aortic valve diameter (mm)	6.96 ± 0.92	6.90 (5.10–9.30)
	Pulmonary valve diameter (mm)	8.89 ± 1.08	8.80 (6.40–11.90)
	RV area, systole (mm^2^)	153.49 ± 41.65	151.00 (78.00–321.00)
	RV area, diastole (mm^2^)	247.61 ± 62.25	243.00 (124.00–458.00)
	RV fractional shortening	0.43 ± 0.12	0.44 (−0.11–0.93)
	LV area, systole (mm^2^)	140.72 ± 43.77	136.00 (47.00–238.00)
	LV area, diastole (mm^2^)	270.95 ± 60.75	263.50 (157.00–427.00)
	LV fractional shortening	0.46 ± 0.16	0.48 (−0.59–0.71)
	RV diameter, systole (mm)	7.98 ± 1.92	7.85 (2.90–11.90)
	RV diameter, diastole (mm)	14.95 ± 10.02	14.05 (7.20–108.00)
	LV diameter, systole (mm)	5.70 ± 1.97	5.45 (2.30–11.90)
	LV diameter, diastole (mm)	10.75 ± 2.71	10.70 (2.20–17.10)
	Interventricular septum thickness (mm)	5.80 ± 1.59	5.65 (3.70–16.70)
	RV wall thickness (mm)	6.57 ± 1.52	6.40 (3.70–12.40)
	LV wall thickness (mm)	7.17 ± 1.70	6.90 (3.60–14.00)
	Right atrial area (mm^2^)	286.74 ± 70.80	276.00 (173.00–584.00)
	Left atrial area (mm^2^)	239.67 ± 44.95	241.00 (131.00–364.00)
	Right atrial diameter (mm)	18.46 ± 2.34	18.20 (13.20–25.90)
	Left atrial diameter (mm)	15.09 ± 1.92	14.90 (9.30–21.10)
	Foramen ovale diameter (mm)	7.20 ± 1.37	7.10 (4.00–11.20)
Doppler	Umbilical artery PI	1.63 ± 7.65	0.83 (0.54–75.00)
	Middle cerebral artery PI	1.53 ± 0.37	1.49 (0.89–2.79)
	Uterine artery PI	0.88 ± 0.43	0.79 (0.37–3.52)

BPD: biparietal diameter; HC: head circumference; OFD: occipito-frontal diameter; AC: abdominal circumference; HL: humeral length; FL: femur length; RV: right ventricle; LV: left ventricle; PI: pulsatility index.

**Table 3:** Paired prenatal ultrasound and postnatal measurements, with inter-measurement differences.

Variable	Postnatal (Neonatal) Mean ± SD	Prenatal (Ultrasound) Mean ± SD	*p*	r	Difference Mean ± SD	Median	Min	Max
Weight (g)	3178.88 ± 359.48	3201.60 ± 399.70	0.335	0.826	−22.71 ± 227.04	−44.77	−780.61	424.72
Abdominal skinfold (mm)	4.32 ± 1.13	3.88 ± 0.85	<0.001	0.458	0.44 ± 1.06	0.40	−2.20	4.90
Femoral skinfold (mm)	8.07 ± 2.40	3.39 ± 0.94	<0.001	0.453	4.68 ± 2.14	4.35	1.00	10.40
Humeral skinfold (mm)	4.86 ± 1.14	3.85 ± 1.18	<0.001	0.324	1.01 ± 1.35	0.80	−3.30	5.20
Head circumference (mm)	348.24 ± 13.96	331.52 ± 16.09	<0.001	0.646	16.73 ± 12.79	17.00	−5.80	103.10
Abdominal circumference (mm)	316.44 ± 19.61	334.57 ± 19.20	<0.001	0.511	−18.13 ± 19.19	−17.70	−67.60	43.50
Thoracic circumference (mm)	330.67 ± 15.01	272.46 ± 27.45	<0.001	0.160	58.21 ± 29.09	55.95	−26.90	224.50
Umbilical-cord entry diameter (mm)	3.54 ± 0.68	7.06 ± 1.52	<0.001	0.224	−3.52 ± 1.52	−3.40	−7.50	−0.70

Difference = postnatal or actual value minus prenatal value; r: Pearson correlation coefficient.

**Table 4:** Factors associated with the discrepancy between actual birth weight and Hadlock-estimated fetal weight.

Group	Median Difference (g)	Lower Limit	Upper Limit	*p*
Cesarean scar count	Cesarean scar count	Cesarean scar count	Cesarean scar count	Cesarean scar count
0–1 (*n* = 63)	−28.2	−544.5	387.7	0.717
≥2 (*n* = 31)	−67.7	−780.6	424.7	
Fetal presentation	Fetal presentation	Fetal presentation	Fetal presentation	Fetal presentation
Cephalic (*n* = 87)	−44.3	−780.6	424.7	0.621
Non-cephalic (*n* = 7)	−122.1	−382.0	290.3	
Placental location	Placental location	Placental location	Placental location	Placental location
Anterior (*n* = 34)	−85.5	−780.6	335.5	0.023
Posterior/fundal (*n* = 60)	1.6	−544.5	424.7	

Values are observed medians, minima, and maxima. *p* values are from the Mann-Whitney U test.

**Table 5:** Regression equations and apparent in-sample characteristics of the developed models.

Model	Equation	R^2^	Mean (g)	SD (g)	Lower Limit (g)	Upper Limit (g)
Actual (neonatal) birth weight	-	-	3178.88	359.48	2230.00	4110.00
Hadlock-estimated fetal weight	EFW = 10^(1.3596 + 0.0064 × HC + 0.0424 × AC + 0.174 × FL + 0.00061 × BPD × AC − 0.00386 × AC × FL)	0.597	3201.60	399.70	2187.59	4257.56
Model 1	y = −7059.05 + 14.417 × AC + 32.226 × FL + 4.861 × HC + 16.277 × BPD	0.994	3201.60	398.52	2107.65	4152.63
Model 2	y = −236.92 + 10.940 × AC + 29.229 × BPD + 15.150 × FL	0.710	3178.88	302.80	2424.87	3908.40
Model 3	y = −5523.29 + 91.401 × THO_C + 92.426 × LENGTH + 46.183 × HUM_SKIN + 25.507 × AC + 16.718 × FEM_SKIN	0.822	3178.88	325.87	2217.24	4015.36
Model 4	y = −4262.33 + 108.344 × THO_C + 23.569 × FEM_SKIN + 79.919 × HC + 19.646 × HUM_SKIN + 24.957 × AC	0.732	3178.88	307.54	2504.61	3953.67
Model 5	y = −4055.58 + 17.473 × FUN_PUB + 26.011 × BPD + 7.953 × AC + 1.006 × RA_SURF − 66.092 × MAT_ABD_SKIN + 16.514 × FL + 29.534 × FEM_SKIN + 30.932 × AO_D	0.783	3178.88	318.09	2379.15	3955.35
Model 6	y = −3969.78 + 5.710 × FUN_PUB + 27.222 × BPD + 8.783 × AC + 1.005 × RA_SURF + 16.055 × FL + 20.302 × FEM_SKIN	0.746	3178.88	310.41	2401.10	3940.89

y: predicted weight (g); EFW: estimated fetal weight; HC: head circumference; AC: abdominal circumference; FL: femur length; BPD: biparietal diameter; THO_C: thoracic circumference; HUM_SKIN: humeral skinfold thickness; FEM_SKIN: femoral skinfold thickness; FUN_PUB: symphysis-fundal height; RA_SURF: right atrial area; MAT_ABD_SKIN: maternal abdominal skinfold thickness; AO_D: aortic valve diameter. All coefficients are unstandardized linear-regression coefficients; SD: standard deviation. For the Hadlock equation, BPD, HC, AC, and FL are entered in centimeters, and EFW is expressed in grams.

**Table 6:** Apparent and internally validated predictive performance of Hadlock and the prenatal birth-weight prediction models (Models 2, 5, and 6).

Model	Apparent R^2^ (95% CI)	Optimism-Corrected R^2^	Repeated 10-Fold CV R^2^	MAE, g (95% CI)	RMSE, g (95% CI)	MAPE (%)	Within ±10%
Hadlock	0.597 (0.352–0.735)	NA	NA	181.0 (154.8–208.9)	227.0 (194.0–263.2)	5.67	89.4%
Model 2	0.710 (0.574–0.799)	0.693	0.683	150.1 (126.0–175.7)	192.7 (164.8–220.7)	4.69	91.5%
Model 5	0.783 (0.682–0.845)	0.739	0.725	133.2 (113.4–153.6)	166.6 (143.3–189.6)	4.13	96.8%
Model 6	0.746 (0.625–0.821)	0.713	0.699	146.1 (125.1–168.3)	180.3 (155.5–205.6)	4.57	93.6%

CI: confidence interval; CV: cross-validation; MAE: mean absolute error; RMSE: root mean squared error; MAPE: mean absolute percentage error; NA, not applicable.

## Data Availability

The datasets generated during the current study are available from the corresponding author upon reasonable request.

## Supplementary Materials

The following supporting information can be downloaded at https://www.mdpi.com/article/10.3390/biomedicines14092122/s1, Table S1. Univariable correlations of actual birth weight and Hadlock-estimated fetal weight with candidate predictors; Table S2. Pairwise correlations between postnatal anthropometry and prenatal ultrasonographic measurements.
