# Supplementary material for: Development of Novel Prenatal Prediction Models for Birth-Weight Estimation in Low-Risk Third-Trimester Pregnancies
Source: Biomedicines. 2026 Sep 19;14(9):2122. doi: 10.3390/biomedicines14092122 (PMC13604655; doi:10.3390/biomedicines14092122)
Supplement: Supplementary file 1 [file biomedicines-14-02122-s001.zip › biomedicines-4507072-supplementary.pdf]

Supplementary Material

Table S1. Univariable correlations of actual birth weight and Hadlock-estimated fetal weight with candidate predictors.

Category	Variable	Hadlock EFW (r)	Actual birth weight (r)
Neonatal measurements	Abdominal skinfold	0.357 (<0.001)	0.335 (<0.001)
	Femoral skinfold	0.420 (<0.001)	0.465 (<0.001)
	Humeral skinfold	0.337 (<0.001)	0.404 (<0.001)
	Head circumference	0.598 (<0.001)	0.646 (<0.001)
	Abdominal circumference	0.557 (<0.001)	0.656 (<0.001)
	Thoracic circumference	0.677 (<0.001)	0.760 (<0.001)
	Umbilical cord entry diameter	0.116 (0.265)	0.054 (0.604)
Head	BPD	0.625 (<0.001)	0.605 (<0.001)
	HC	0.544 (<0.001)	0.442 (<0.001)
	OFD	0.470 (<0.001)	0.451 (<0.001)
Abdomen	AC	0.883 (<0.001)	0.747 (<0.001)
	Abdominal skinfold	0.305 (0.003)	0.355 (<0.001)
	Abdominal AP diameter	0.598 (<0.001)	0.492 (<0.001)
	Abdominal transverse diameter	0.576 (<0.001)	0.513 (<0.001)
Humerus	HL	0.379 (<0.001)	0.375 (<0.001)
	Humeral skinfold	0.329 (0.001)	0.359 (<0.001)
Femur	FL	0.560 (<0.001)	0.409 (<0.001)
	Femoral skinfold	0.291 (0.004)	0.312 (0.002)
Umbilical cord	Cross-sectional area	0.183 (0.078)	0.072 (0.488)
	Wharton's jelly area	0.122 (0.243)	0.023 (0.829)
	Entry diameter	0.377 (<0.001)	0.363 (<0.001)
Placenta	Thickness	0.073 (0.484)	0.196 (0.058)
Thorax	Circumference	0.209 (0.043)	0.227 (0.028)
	AP diameter	0.364 (<0.001)	0.303 (0.003)
	Transverse diameter	0.290 (0.005)	0.270 (0.008)

Heart	Heart angle	0.006 (0.954)	0.054 (0.605)
	Cardiac circumference	0.392 (<0.001)	0.423 (<0.001)
	Tricuspid valve diameter	0.159 (0.125)	0.198 (0.055)
	Mitral valve diameter	0.114 (0.273)	0.219 (0.034)
	Aortic valve diameter	0.245 (0.017)	0.272 (0.008)
	Pulmonary valve diameter	0.203 (0.050)	0.295 (0.004)
	RV area, systole	0.152 (0.143)	0.175 (0.091)
	RV area, diastole	0.289 (0.005)	0.346 (<0.001)
	RV fractional shortening	-0.018 (0.866)	0.087 (0.403)
	LV area, systole	0.129 (0.215)	0.186 (0.073)
	LV area, diastole	0.159 (0.126)	0.200 (0.053)
	LV fractional shortening	0.219 (0.034)	0.251 (0.015)
	RV diameter, systole	0.056 (0.590)	0.008 (0.941)
	RV diameter, diastole	0.027 (0.793)	0.038 (0.716)
	LV diameter, systole	0.057 (0.585)	0.034 (0.742)
	LV diameter, diastole	0.178 (0.086)	0.154 (0.139)
	Interventricular septum	0.220 (0.033)	0.342 (<0.001)
	RV wall thickness	0.207 (0.045)	0.273 (0.008)
	LV wall thickness	-0.013 (0.904)	0.011 (0.918)
	Right atrial area	0.393 (<0.001)	0.485 (<0.001)
	Left atrial area	0.343 (<0.001)	0.427 (<0.001)
	Right atrial diameter	0.364 (<0.001)	0.387 (<0.001)
	Left atrial diameter	0.276 (0.007)	0.327 (0.001)
	Foramen ovale diameter	0.309 (0.002)	0.376 (<0.001)
Doppler	Umbilical artery PI	-0.012 (0.909)	-0.047 (0.655)
	MCA PI	0.181 (0.081)	-0.023 (0.822)
	Uterine artery PI	0.006 (0.955)	0.056 (0.589)
Maternal / conventional	Symphysis-fundal height	0.545 (<0.001)	0.488 (<0.001)
	Maternal abdominal skinfold	0.140 (0.179)	-0.036 (0.733)
	BMI	0.225 (0.029)	0.173 (0.096)
	Gestational weight gain	0.103 (0.335)	0.209 (0.049)

EFW, estimated fetal weight. Values are Pearson correlation coefficients.

Table S2. Pairwise correlations between postnatal anthropometry and prenatal ultrasonographic measurements.

		Neonatal measurements	Neonatal measurements	Neonatal measurements	Neonatal measurements	Neonatal measurements	Neonatal measurements	Neonatal measurements	Prenatal ultrasound measurements	Prenatal ultrasound measurements	Prenatal ultrasound measurements	Prenatal ultrasound measurements	Prenatal ultrasound measurements	Prenatal ultrasound measurements
		Abd. skin	Fem. skin	Hum. skin	HC	AC	THO_C	Umb. entry	Abd. skin	Fem. skin	Hum. skin	HC	AC	THO_C
Neonatal measurements	Femoral skinfold	0.61												
Neonatal measurements	Humeral skinfold	0.26	0.54											
Neonatal measurements	Head circumference	0.14	0.26	0.32										
Neonatal measurements	Abdominal circumference	0.18	0.35	0.27	0.44									
Neonatal measurements	Thoracic circumference	0.23	0.32	0.27	0.48	0.68								
Neonatal measurements	Umbilical cord entry	0.10	0.06	-0.01	0.07	0.03	0.09							
Prenatal ultrasound measurements	Abdominal skinfold	0.46	0.42	0.24	0.20	0.30	0.31	0.11						
Prenatal ultrasound measurements	Femoral skinfold	0.35	0.45	0.31	0.07	0.10	0.21	0.03	0.39					
Prenatal ultrasound	Humeral skinfold	0.25	0.30	0.32	0.18	0.16	0.23	-0.11	0.49	0.49				

measurements														
Prenatal ultrasound measurements	Head circumference	0.12	0.12	0.21	0.65	0.26	0.36	-0.02	0.09	-0.03	0.20			
Prenatal ultrasound measurements	Abdominal circumference	0.38	0.44	0.33	0.40	0.51	0.58	0.13	0.27	0.35	0.24	0.28		
Prenatal ultrasound measurements	Thoracic circumference	0.30	0.21	0.01	0.12	0.18	0.16	0.01	0.09	0.08	0.06	0.12	0.19	
Prenatal ultrasound measurements	Umbilical cord entry	0.28	0.37	0.24	0.28	0.22	0.25	0.22	0.20	0.12	0.08	0.25	0.39	-0.08

Values are Pearson correlation coefficients; blank cells represent the upper triangle or self-correlations.
